# Supplementary figures and images for: Supplementary education can improve the rate of adequate bowel preparation in outpatients: A systematic review and meta-analysis based on randomized controlled trials
Source: PLoS One. 2022 Apr 21;17(4):e0266780. doi: 10.1371/journal.pone.0266780 (PMC9023061; doi:10.1371/journal.pone.0266780)

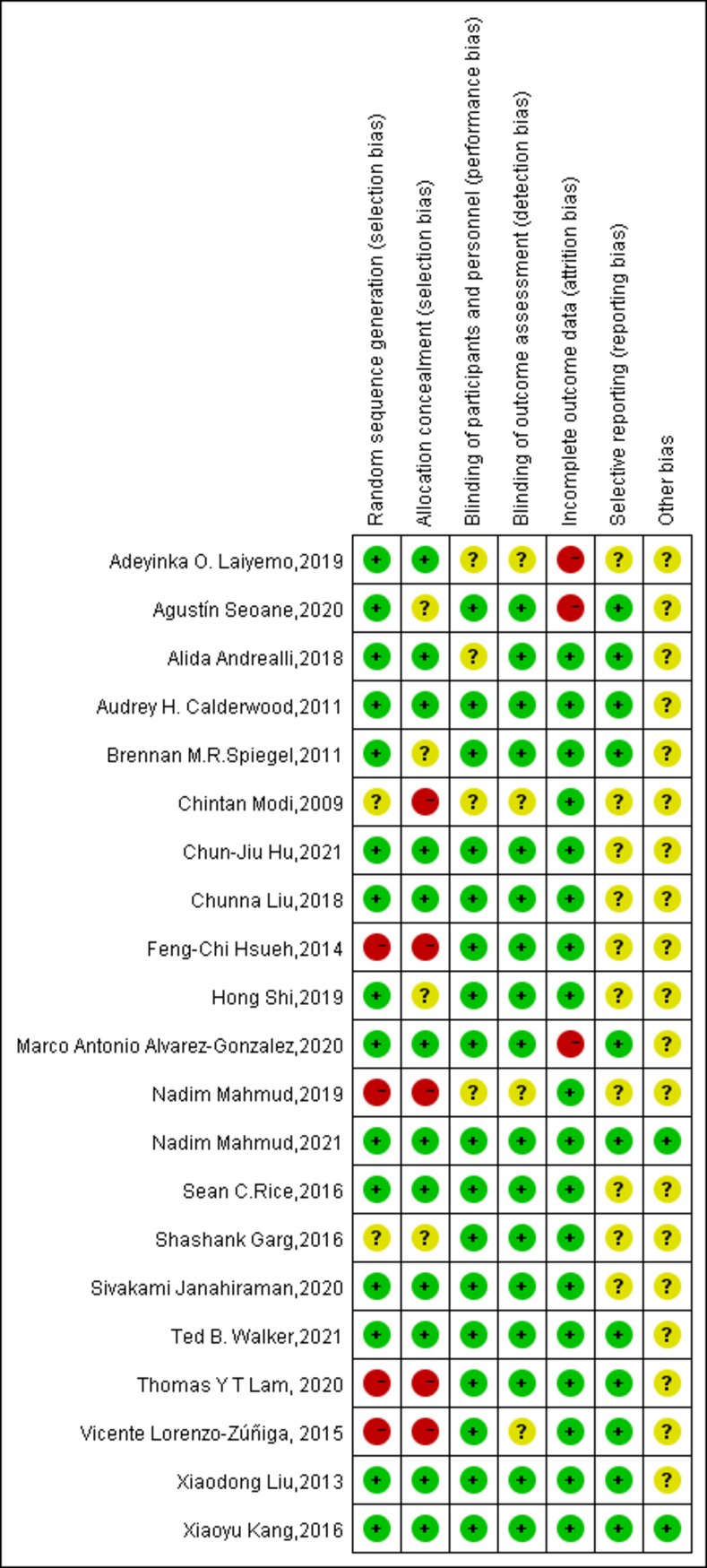

Supplement: S1 Fig — (TIF) [file pone.0266780.s002.tif]

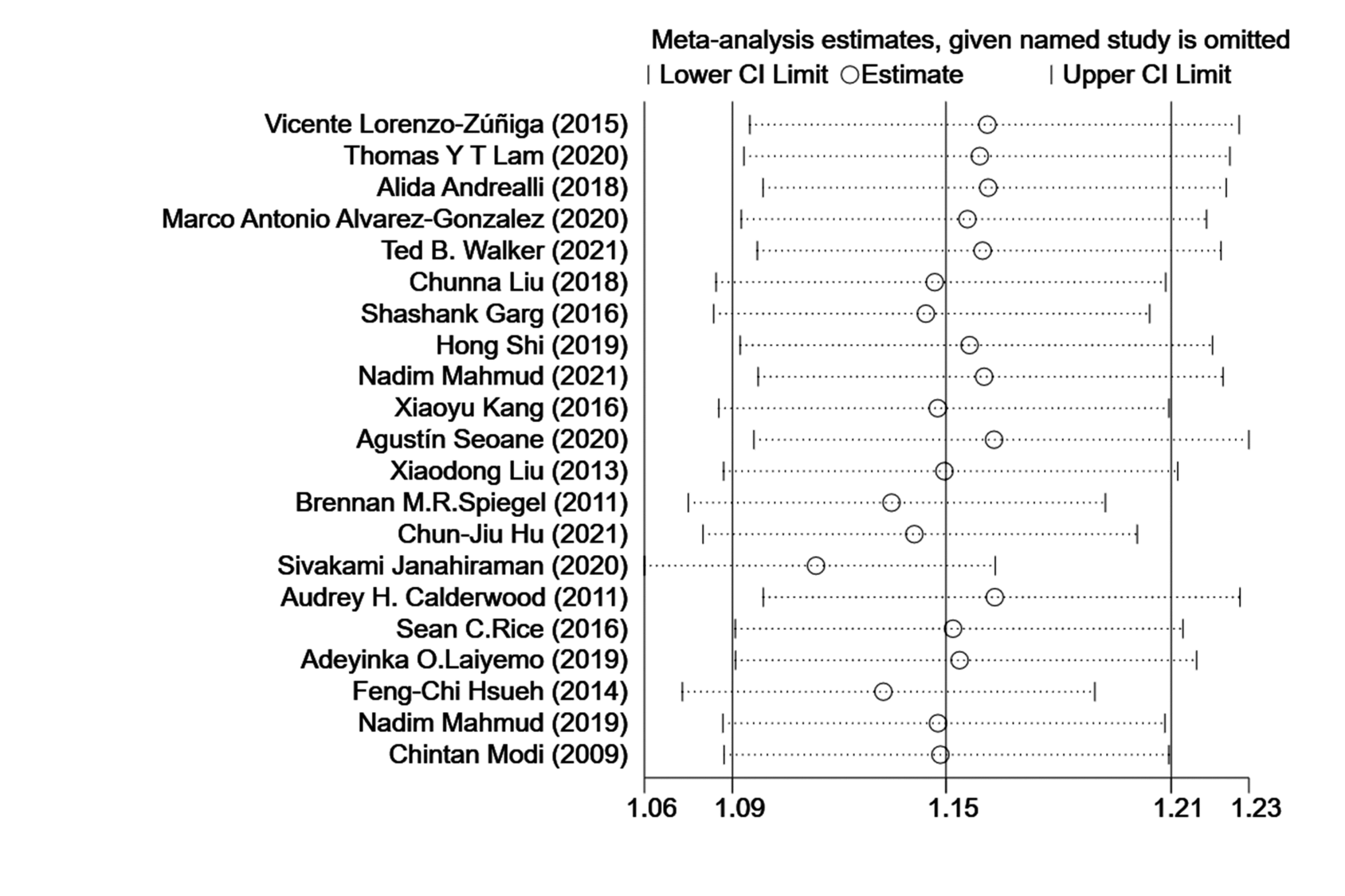

Supplement: S2 Fig — (TIF) [file pone.0266780.s003.tif]

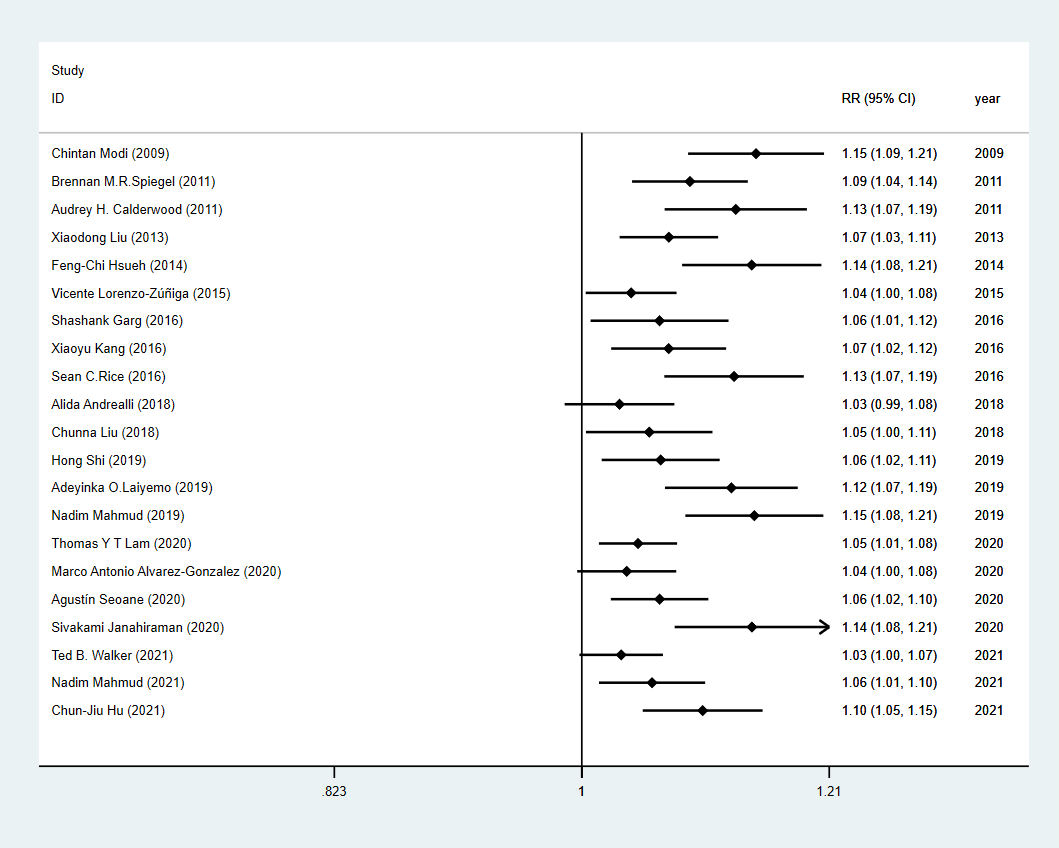

Supplement: S3 Fig — (TIF) [file pone.0266780.s004.tif]

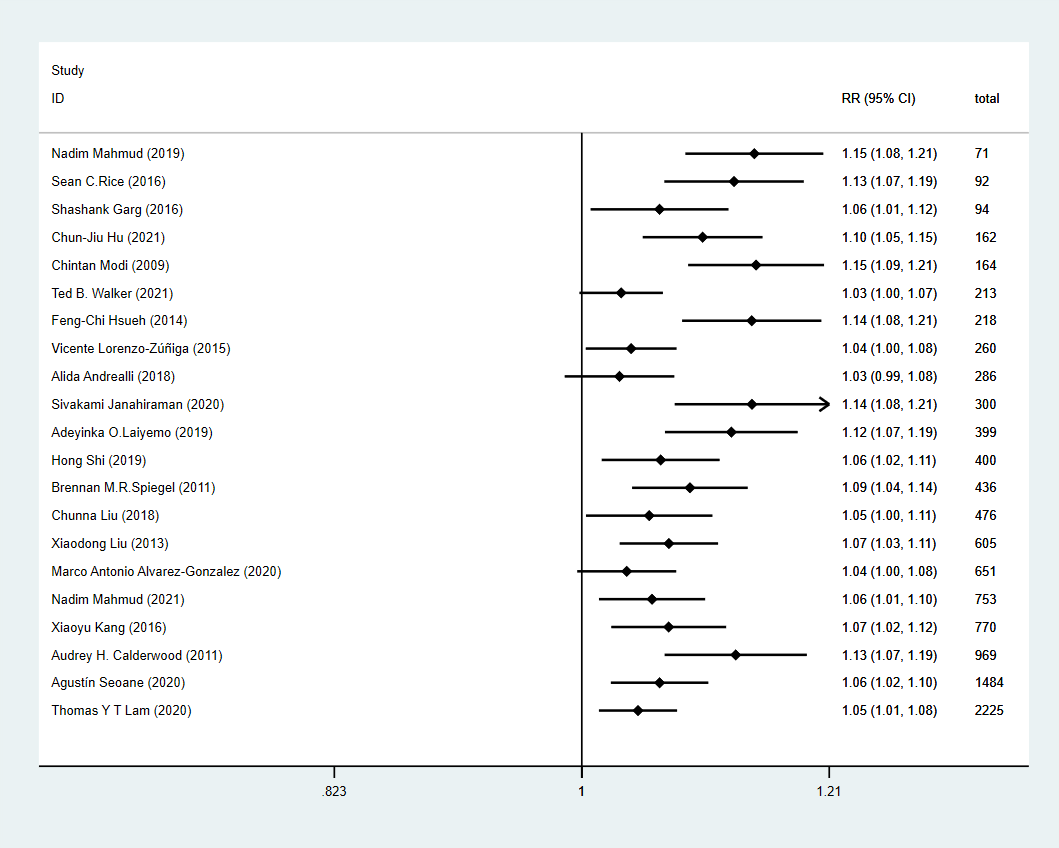

Supplement: S4 Fig — (TIF) [file pone.0266780.s005.tif]

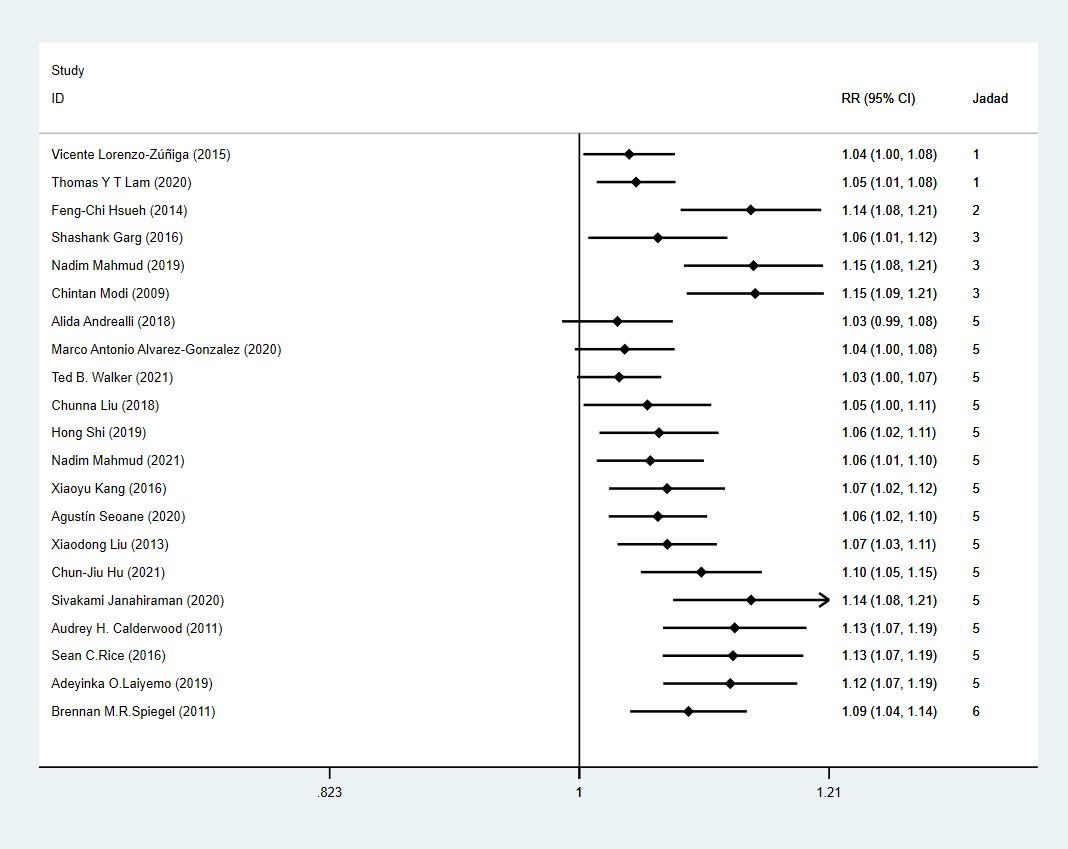

Supplement: S5 Fig — (TIF) [file pone.0266780.s006.tif]

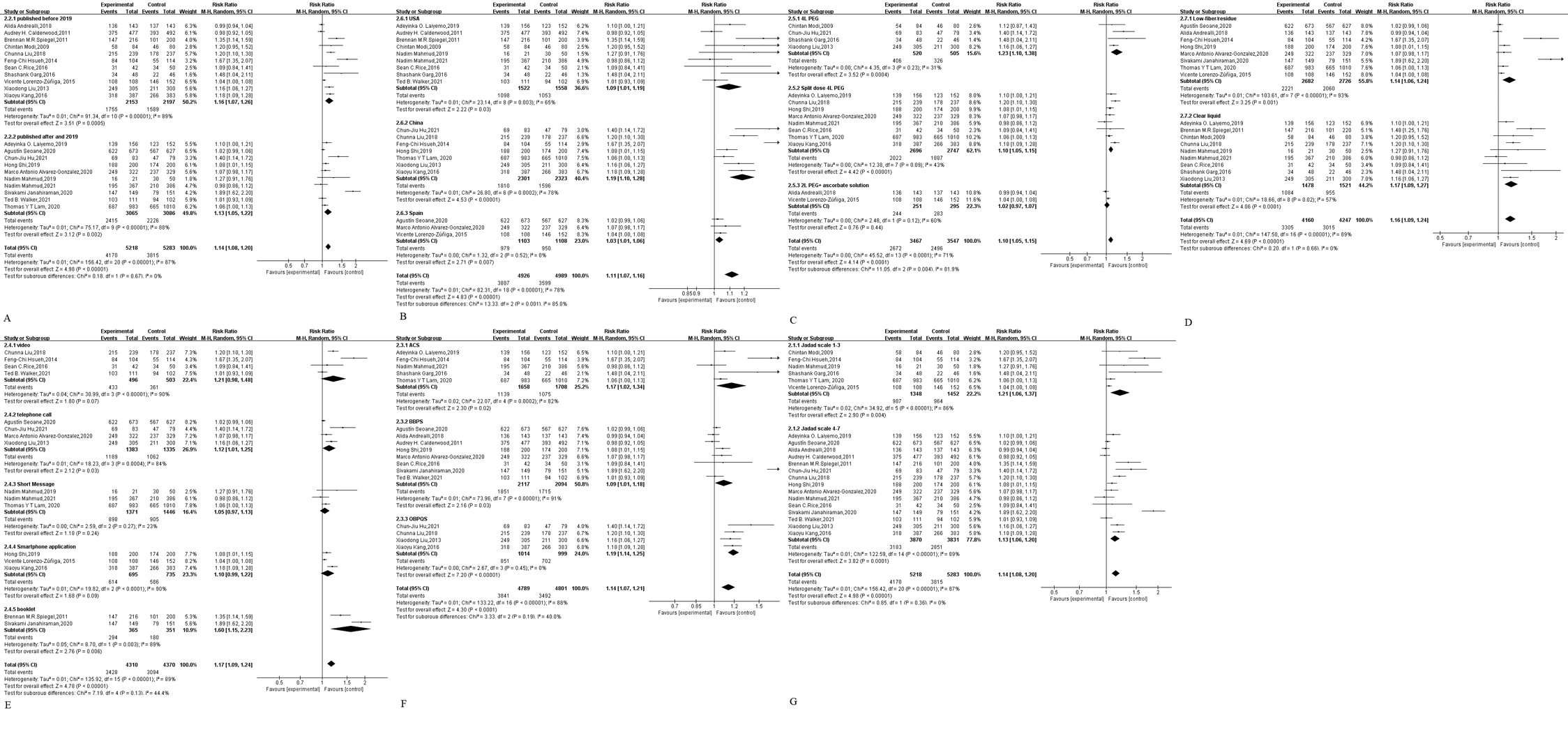

Supplement: S6 Fig — Sensitivity analysis comparing the effects of supplementary education combined with traditional education and traditional education alone on the adequate bowel preparation rate based on (A) publication year, (B) country, (C) bowel preparation regimen, (D) diet restriction, (E) supplementary education method, (F) quality evaluation scale and (G) Jadad score. (TIF) [file pone.0266780.s007.tif]

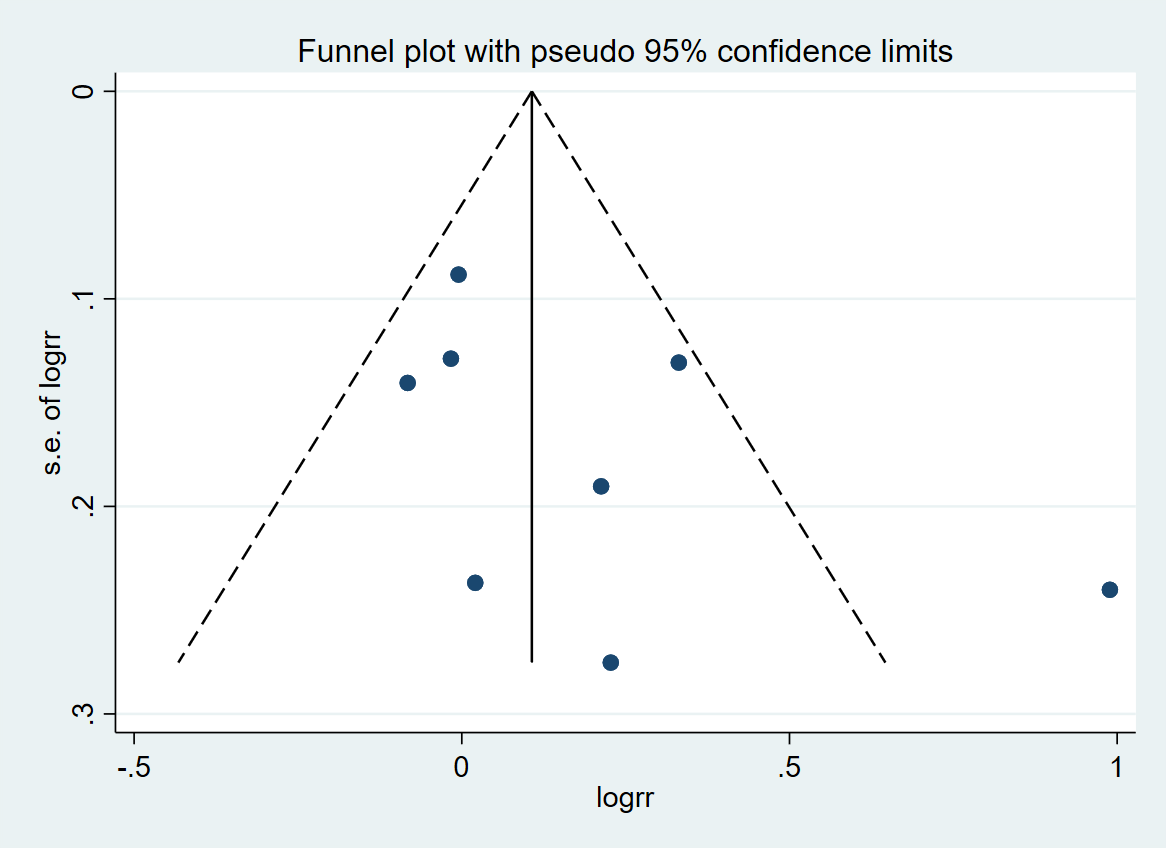

Supplement: S7 Fig — (TIF) [file pone.0266780.s008.tif]

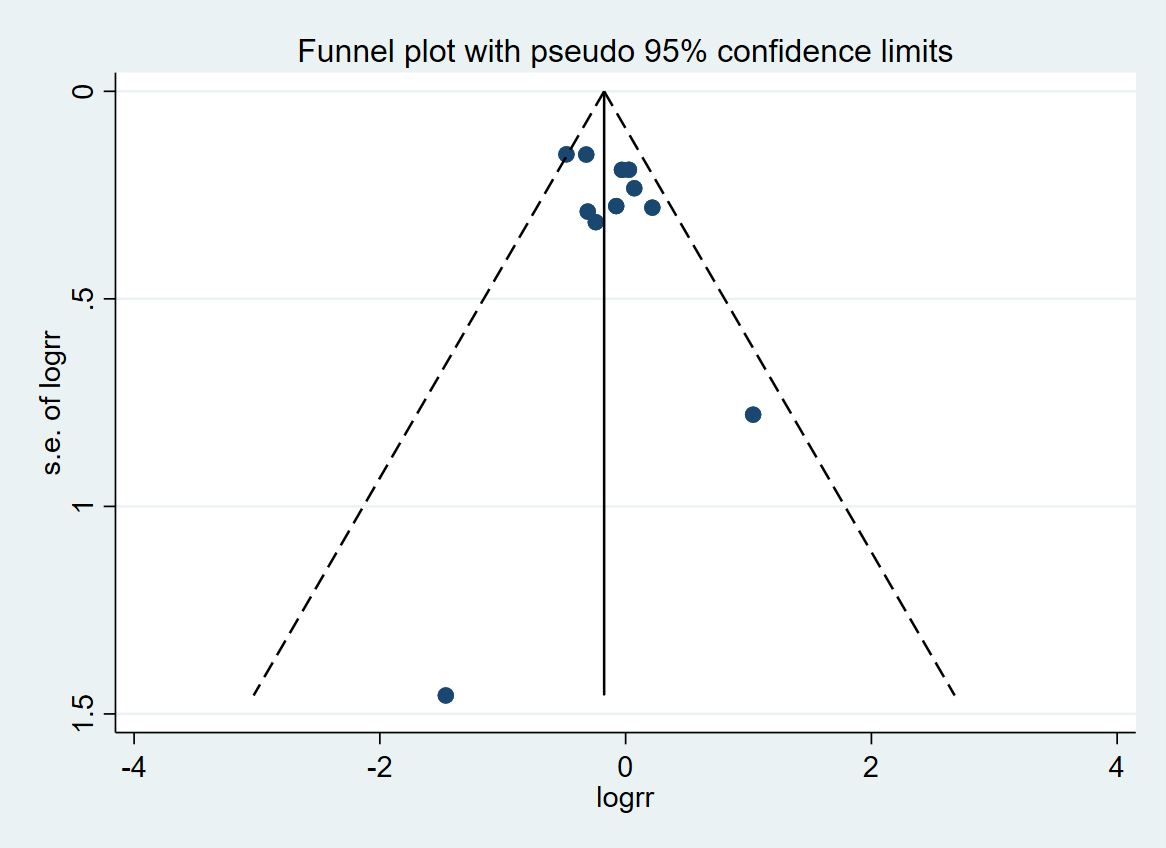

Supplement: S8 Fig — (TIF) [file pone.0266780.s009.tif]

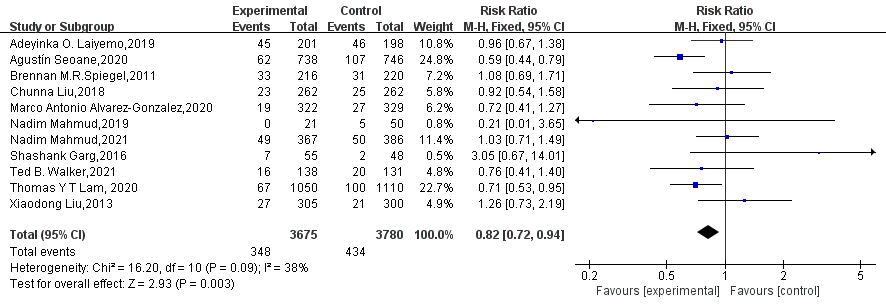

Supplement: S9 Fig — (TIF) [file pone.0266780.s010.tif]
