# Supplementary material for: Supplementary education can improve the rate of adequate bowel preparation in outpatients: A systematic review and meta-analysis based on randomized controlled trials
Source: PLoS One. 2022 Apr 21;17(4):e0266780. doi: 10.1371/journal.pone.0266780 (PMC9023061; doi:10.1371/journal.pone.0266780)
Supplement: S1 File — (DOCX) [file pone.0266780.s011.docx]

**Identification of studies via databases and registers**

Records removed *before screening*: Duplicate records removed (n = 361)

Records identified from: Web of Science (n= 1142), PubMed (n= 228) and Cochrane Library (n= 692)

**Identification**

Records screened

(n = 1701)

Records excluded after title and abstract (n = 1613)

Reports sought for retrieval

(n = 88)

Conference abstracts and posters (n = 46)

**Screening**

Reports assessed for eligibility

(n = 42)

Reports excluded: duplicate (n= 5), Nonrandomized controlled trial (n= 7), Nonclinical research (n= 5), No standard of care educational materials in the intervention group (n= 3) and lack of outcome indicators (n= 1)

Studies included in review

(n = 21)

**Included**

*Consider, if feasible to do so, reporting the number of records identified from each database or register searched (rather than the total number across all databases/registers).

**If automation tools were used, indicate how many records were excluded by a human and how many were excluded by automation tools.

*From:*  Page MJ, McKenzie JE, Bossuyt PM, Boutron I, Hoffmann TC, Mulrow CD, et al. The PRISMA 2020 statement: an updated guideline for reporting systematic reviews. BMJ 2021;372:n71. doi: 10.1136/bmj.n71

For more information, visit: <http://www.prisma-statement.org/>
