# Supplementary material for: Supplementary education can improve the rate of adequate bowel preparation in outpatients: A systematic review and meta-analysis based on randomized controlled trials
Source: PLoS One. 2022 Apr 21;17(4):e0266780. doi: 10.1371/journal.pone.0266780 (PMC9023061; doi:10.1371/journal.pone.0266780)
Supplement: S1 Table — (DOCX) [file pone.0266780.s012.docx]

**Supplementary table 1** Quality evaluation based on the Jadad scale

| study | Randomization | | | concealment of allocation | | | double blinding | | | withdraws and dropouts | | score |
| --- | --- | --- | --- | --- | --- | --- | --- | --- | --- | --- | --- | --- |
|  | 0 | 1 | 2 | 0 | 1 | 2 | 0 | 1 | 2 | 0 | 1 |  |
| Vicente Lorenzo-Zúñiga, 2015[14] | + |  |  | + |  |  | + |  |  |  | + | 1 |
| Thomas Y T Lam, 2020[19] | + |  |  | + |  |  | + |  |  |  | + | 1 |
| Alida Andrealli,2018[34] |  |  | + |  |  | + | + |  |  |  | + | 5 |
| Marco Antonio Alvarez-Gonzalez,2020[33] |  |  | + |  |  | + | + |  |  |  | + | 5 |
| Ted B. Walker,2021[11] |  |  | + |  |  | + | + |  |  |  | + | 5 |
| Chunna Liu,2018[12] |  |  | + |  |  | + | + |  |  |  | + | 5 |
| Shashank Garg,2016[32] |  | + |  |  | + |  | + |  |  |  | + | 3 |
| Hong Shi,2019[35] |  |  | + |  |  | + | + |  |  |  | + | 5 |
| Nadim Mahmud,2021[20] |  |  | + |  |  | + | + |  |  |  | + | 5 |
| Xiaoyu Kang,2016[15] |  |  | + |  |  | + | + |  |  |  | + | 5 |
| Agustín Seoane,2020[17] |  |  | + |  |  | + | + |  |  |  | + | 5 |
| Xiaodong Liu,2013[7] |  |  | + |  |  | + | + |  |  |  | + | 5 |
| Brennan M.R. Spiegel,2011[9] |  |  | + |  |  | + |  | + |  |  | + | 6 |
| Chun-Jiu Hu,2021[18] |  |  | + |  |  | + | + |  |  |  | + | 5 |
| Sivakami Janahiraman,2020[10] |  |  | + |  |  | + | + |  |  |  | + | 5 |
| Audrey H. Calderwood,2011[31] |  |  | + |  |  | + | + |  |  |  | + | 5 |
| Sean C. Rice,2016[13] |  |  | + |  |  | + | + |  |  |  | + | 5 |
| Adeyinka O. Laiyemo,2019[30] |  |  | + |  |  | + | + |  |  |  | + | 5 |
| Feng-Chi Hsueh,2014[29] | + |  |  | + |  |  |  | + |  |  | + | 2 |
| Nadim Mahmud,2019[21] |  |  | + | + |  |  | + |  |  |  | + | 3 |
| Chintan Modi,2009[28] |  | + |  |  | + |  | + |  |  |  | + | 3 |
